# Assessment of EGFR/HER2 dimerization by FRET-FLIM utilizing Alexa-conjugated secondary antibodies in relation to targeted therapies in cancers

**DOI:** 10.18632/oncotarget.313

**Published:** 2011-09-08

**Authors:** Benjamin R. Waterhouse, Merel Gijsen, Paul R. Barber, Iain D.C. Tullis, Borivoj Vojnovic, Anthony Kong

**Affiliations:** ^1^Human Epidermal Growth Factor Receptor Group; Cancer Research UK Molecular Oncology Laboratories, The Weatherall Institute of Molecular Medicine (WIMM), University of Oxford, John Radcliffe Hospital, Oxford, OX3 9DS, UK; ^2^Gray Institute for Radiation Oncology and Biology, University of Oxford, Old Road Campus Research Building, Off Roosevelt Drive, Churchill Hospital, Oxford, OX3 7DQ, UK

**Keywords:** HER (ErbB) receptors, EGFR, HER2 (ErbB2), dimerization, FRET, FLIM

## Abstract

The expression level of the HER family is unreliable as a predictive marker for targeted therapies in cancer. Thus, there is a need to develop other biomarkers, which can be used to accurately select responsive patients for targeted therapies. The HER dimerization status may be more important than HER receptor expression *per se* in determining sensitivity or resistance to a given therapeutic agent. The aim of the study is to develop a FRET assay using dye conjugated secondary antibodies to assess HER receptor dimerization. Using primary antibodies from different species in conjunction with Alexa488 and Alexa546 conjugated secondary antibodies, we validated our EGFR/HER2 dimerization assay in three cell lines, EGFR positive A431 cells as well as HER2 positive breast cell lines BT474 and SKBR3 cells. Finally, we applied our assay to assess EGFR/HER2 dimerization in paraffin embedded cell pellets. Our results show promise for the assay to be applied to tumor samples in order to assess the prognostic significance and predictive value of HER receptor dimerization in various cancers.

## INTRODUCTION

Dysregulation of HER (ErbB) receptors has been implicated in several cancers. For example, EGFR is frequently over-expressed in Head and Neck Squamous Cell Carcinoma (HNSCC) and is correlated with poor disease-free survival and overall survival in these patients [1, 2]. In breast cancer, over-expression and amplification of HER2 occurs in about 15-20% of breast cancer and is predictive of poor outcome in these patients [3-5]. Trastuzumab (Herceptin) was developed to target HER2 proteins and has been shown to increase the survival of patients [6, 7]. However, the response rate for Herceptin monotherapy for advanced breast cancer patients is only around 35% and all responders eventually develop resistance to Herceptin [8]. In lung cancers, EGFR mutations were found to predict an excellent response to EGFR TKIs but these are only found in a small number of patients [9, 10]. Furthermore, such mutations are uncommon in other cancers like HNSCC and may not be predictive of sensitivity to EGFR inhibitors in these cancers [11]. For those cancers without EGFR mutations, it has been shown that the expression levels of EGFR do not predict the success of these drugs and the response rate to these inhibitors remains poor [9, 12]. One reason for this is that the HER receptors are able to form alternative dimers and can therefore compensate the loss of function of one receptor during targeted therapies [13, 14]. Thus, the HER receptor dimerization patterns may be more important than the expression level *per se* in cancer prognosis and prediction of response to targeted therapies [15, 16]. Therefore, the ability to assess the dimerization pairs within tumours could be useful as a prognostic or predictive biomarker for targeted therapies in cancer.

In cell lines co-immunoprecipitation (IP) can be used to make an assessment of HER dimerization. However, this method is not ideal since the two HER proteins assessed may not have interacted with each other directly but through a tertiary protein. Assessment of HER dimerization states in tumours has also been proven to be difficult since the conventional method utilizing IHC can only assess the expression levels and their co-localization of HER receptors. For example, in EGFR and HER2 staining, the percentage of stained cells and their intensity allows us to assess EGFR and HER2 expression in the tumors. However, staining for both receptors in a single sample can only reveal intercellular co-localization but this does not provide a true indication of their dimerization states. The same limitation applies to intensity-based fluorescence/confocal microscopy, which can reveal intracellular co-localization but not definitively demonstrate the existence of HER dimers.

We hypothesized that HER dimerization could be quantified by Förster Resonance Energy Transfer (FRET) after successfully applying the assay to assess EGFR activation states in tissue microarrays [17]. Previously, we conjugated primary antibodies to a pair of fluorophores. However, such experiments often resulted in a waste of large amount of antibodies during the primary antibody conjugation process and thus was not economically viable. Here we propose and test the use of a pair of commercially available fluorophore-labeled secondary antibodies specific for different species of primary to conduct a FRET experiment. This protocol could potentially be applied to assess various HER receptor dimerization and other protein interactions by varying the primary antibodies. Our aim is to apply the assay to assess EGFR/HER2 dimerization in HER2 positive breast tumors as well as EGFR over-expressing tumors like HNSCC. As a proof of principle, we have validated our assay for the dimerization of EGFR and HER2 in 3 cell lines; HER2 positive cell lines BT474 and SKBr3 as well as EGFR positive A431 cells, in relation to ligand stimulation and targeted therapies. We also applied the assay to paraffin-embedded cell pellets to demonstrate the robustness of the assay and the potential clinical application to tissue microarrays (TMA).

## RESULTS

**Figure 1:**
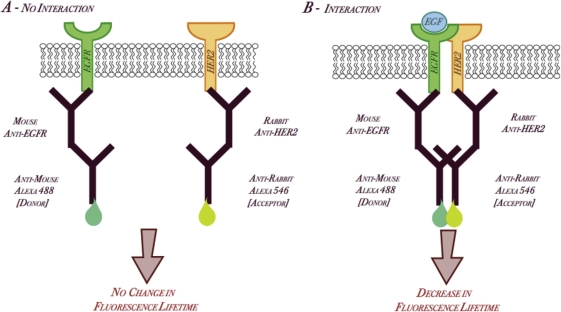
Schematic Diagram of EGFR/HER2 dimerization by FRET utilizing Alexa-conjugated secondary antibodies

Using primary antibodies for EGFR and HER2 from two different species in conjunction with species-specific secondary antibodies conjugated to Alexa488 or Alexa546, we first assessed the dimerization of EGFR and HER2 in fixed cell samples. The schematic diagram is for this labeling is shown in Figure 1. We established that there is an advantage in utilizing the over-expressed receptor as the FRET donor (data not shown). For example, in EGFR over-expressing cell lines where we chose EGFR as the donor, we used a mouse-EGFR antibody with an anti-mouse Alexa488 secondary antibody. HER2 would be the acceptor, so we would use a rabbit-HER2 antibody with an anti-rabbit Alexa546 secondary antibody. It was hypothesized that upon ligand simulation, there would be an increase in EGFR/HER2 dimerization and thus FRET between the two conjugated flurophores, The overlap in the donor (Alexa488) emission spectrum and acceptor (Alexa546) absorption spectrum allows FRET to occur, resulting in a decrease of donor fluorescence lifetime, indicative of EGFR/HER2 dimerization (Figure 1). In HER2 over-expressing cell lines, we used a rabbit-HER2 antibody with an anti-rabbit Alexa488 secondary antibody as donor and a mouse-EGFR antibody with an anti-mouse Alexa546 secondary antibody as acceptor to assess EGFR/HER2 dimerization by FRET.

**Figure 2:**
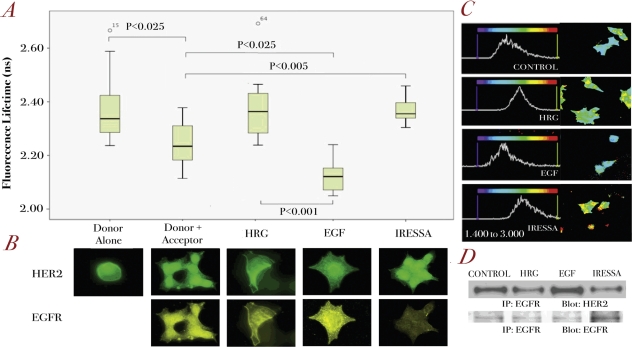
Assessment of EGFR/HER2 dimerization by FRET in BT474 cells **A.** BT474 cells were stimulated with different ligands, EGF and HRG (both 100ng/ml for 10 min) or pre-treated with Iressa 1μM for 1 hour before being fixed for FRET experiments (see Methods). Analysis was performed globally across each group of cells using a bi-exponential fit and interpreting the slower component as the fluorescence lifetime (See Supplementary Figure 1) (n=20 for each condition) **B.** Panels of fluorescence microscopy images are shown for each of these conditions illustrating EGFR (lower panels) and HER2 (upper panels) receptor localization. **C.** As in A, a bi-exponential fit and the slow components were used. Here, fits were performed on a pixel-by-pixel basis and used to generate pseudo-colour fluorescence lifetime images, with a scale from 1.4 ns to 3.0 ns, showing the distribution of fluorescence lifetimes across the cells. The corresponding histograms are generated from the relative frequency of lifetimes across this range thus showing the shifts that occur between treatment conditions. **D.** Co-immunoprecipitation blots for EGFR and HER2 on BT474 cell lysate for the different conditions as described in A.

To show *in-situ* that EGFR/HER2 dimerization could be monitored by our assay, BT474 cells were stimulated with HER ligands, either EGF or heregulin as well as by an EGFR-specific tyrosine kinase inhibitor, Iressa. Firstly, we stained unstimulated BT474 cells with only the primary rabbit-HER2 antibody and the correlating anti-rabbit Alexa488 secondary antibody (as described in Materials and Methods). The lifetime measured in this sample gives us the fluorescence lifetime of Alexa 488, which was shown to have a mean value of approximately 2.35 ns (Figure 2A). When the cells were stained with both the primary antibodies (anti-HER2 and anti-EGFR) as well as the appropriate species-specific labeled secondary antibodies, we found a statistically significant decrease in fluorescence donor lifetime to around 2.25 ns (P<0.025) compared to that with the donor alone (Figure 2A), suggesting the existence of a basal level of EGFR/HER2 dimerization, as confirmed by immunoprecipitation and western blot (Figure 2D).

When BT474 cells were treated with EGF, there was a further decrease in donor lifetime to 2.12 ns which is statistically significant compared to the result from unstimulated cells (P<0.025) (Figure 2A), which correlated with the higher amount of EGFR-HER2 dimerization seen in IP (Figure 2D). As a negative control, BT474 cells were stimulated with heregulin (HRG), which induces dimerization of HER3 and HER4, with their preferred dimerization partner HER2 [18]. Therefore, this might be seen to decrease EGFR/HER2 dimerization (Figure 2A), which was confirmed by IP (Figure 2D). To further demonstrate the specificity of our assay, another negative control was used. EGFR specific tyrosine kinase inhibitor gefitinib (Iressa), which was shown to decrease basal EGFR/HER2 dimerization (Figure 2D), produced an increase in donor fluorescence lifetime when compared to that of untreated cells (P<0.005) (Figure 2A). A visual representation of the distribution of fluorescence lifetimes across typical cells, using a pseudo-colour display, ranging from 1.40 – 3.00 ns, is shown in Figure 2C. The image for EGF treated cells shows almost entirely ‘blue’ pixels compared to the blue/yellow combination seen in the control, or yellow/green combination in HRG and Iressa treated cells. Each image is accompanied by a graphical representation of these lifetimes. In the case of EGF-treated cells, this distribution is seen to shift towards lower lifetimes, compared to the control or HRG and Iressa treated cells.

Figure 2B represents fluorescence images of these cells with HER2 shown in green and EGFR in yellow. As can be seen here, the shortcoming of fluorescence images, and that of confocal images, is that only co-localization of receptors can be suggested rather than true interactions. However, in combination with donor lifetime determinations, we could conclude whether the dimerization of EGFR/HER2 has indeed occurred (Figure 2A).

**Figure 3:**
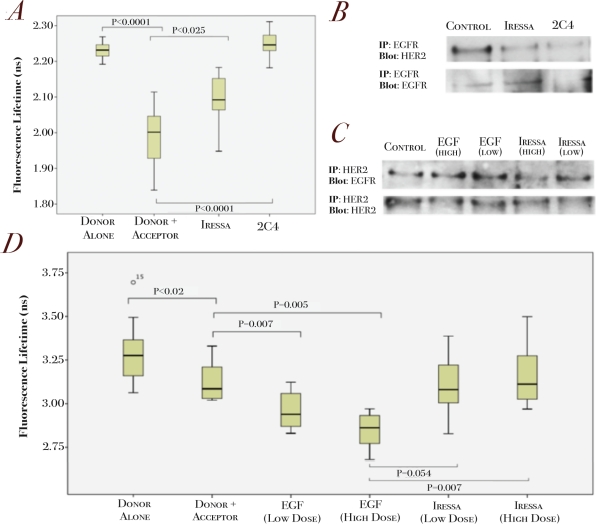
Validation of the EGFR/HER2 dimerization assay in relation to targeted therapies in HER2-positive and EGFR-positive cell lines **A.** SKBR3 cells were pre-treated with Iressa (1uM, 1h) and pertuzumab (1μg/ml, 1h) before being fixed for FRET experiments (see Methods). Changes in fluorescence lifetime of the different conditions are shown (n=15-20 for each condition). **B.** SKBR3 cells were treated with either 1 μM Iressa or 1μg/ml pertuzumab (2C4) for 1 hour before subjected to immunoprecipitation for EGFR and blotted for EGFR (lower panels) and HER2 (upper panels). **C.** A431 cells were stimulated with 500 ng/ml (HIGH) or 100 ng/ml (LOW) EGF for 10 min or pre-treated with Iressa treatment for 1 hour at 5 μM (HIGH) or 1 μM (LOW) before subjected to immunoprecipitation for HER2 and blotted for EGFR (upper panels) and HER2 (lower panels). **D.** A431 cells were treated with the same conditions as C before being fixed for FRET experiments. Dose dependent changes in lifetime for EGF and Iressa treated A431 cells are shown.

We also validated our assay in another HER2 positive breast cell lines SKBr3 cells treated with different targeted therapies (Figure 3A). We showed that Iressa decreased basal EGFR/HER2 dimerization as shown by an increase of donor lifetime compared to basal level (Figure 3A and 3B). Pertuzumab specifically blocks dimerization of HER2 by binding to domain II and sterically preventing association with other receptors [19], and thus is an ideal control to validate our assay. As seen in figure 3A, and supported by immunoprecipitation (Figure 3B), 1 hour pre-treatment with Pertuzumab resulted in an increase in donor fluorescence lifetime, returning the lifetime to be in line with the donor alone control lifetime. This implies that dimerization is prevented between EGFR and HER2, which is consistent with the mode of action.

In the EGFR positive A431 cell line, a mouse anti-EGFR antibody was used as the primary antibody, with specific anti-mouse secondary antibody coupled to Alexa488 and anti-rabbit HER2 primary antibody coupled with an Alexa546-secondary anti-rabbit antibody (Figure 1). Figure 3D showed that A431 cells stained with donor and acceptor compared to donor alone gave significantly lower lifetimes, indicative of the basal EGFR/HER2 dimerization in these cells (Figure 3C and 3D). To show that our assay is sensitive to detect the changes in EGFR/HER2 dimerization, we stimulated the cells with increased doses of EGF and assessed the donor lifetimes. Figure 3D shows that application of a low dose of EGF produces a smaller decrease in donor lifetime compared to the higher dose. The converse was shown to be true with increasing doses of Iressa, which decreased EGFR/HER2 dimerization (Figure 3D). These changes in fluorescence lifetime correspond to dimerization as assessed by immunoprecipitation (Figure 3C), thus demonstrating the sensitivity of our assay in detecting changes in EGFR/HER2 dimerization in response to different doses of ligand stimulation and drug treatment.

**Figure 4:**
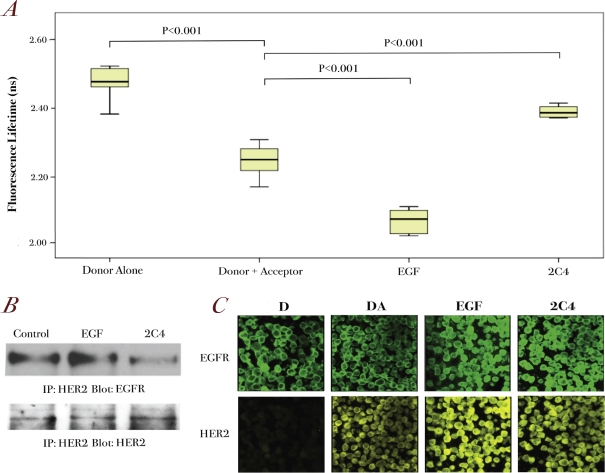
Further validation of the EGFR/HER2 dimerization assay in paraffin-embedded cell pellets **A.** A431 cells were stimulated with 100 ng/ml EGF for 10 min or pre-treated with1 μg/ml pertuzumab for 1 hour before being paraffin-embedded for FRET experiments (see Methods). The fluorescence lifetime changes for each condition are shown (n=12-15 for each condition) **B.** Co-immunoprecipitation of EGFR and HER2 on A431 cells after the same treatment as described in A. **C.** Fluorescence intensity images of EGFR (upper panels) and HER2 (lower panels) for A431 cell pellets pre-treated with 100 ng/ml EGF for 10 min or 1μg/ml pertuzumab for 1 hour.

To assess whether we could apply the assay to assess EGFR/HER2 dimerization in TMAs, we needed to demonstrate its sensitivity to detect EGFR/HER2 dimerization changes in paraffin-embedded cell pellets. Thus, we applied the assay to A431 paraffin-embedded cell pellets of different pre-treatment conditions. We performed the assay using anti-EGFR antibody as primary antibody with the correct secondary labeled antibody as the FRET donor. The decrease in fluorescence lifetime for the donor and acceptor control sample suggested a degree of basal dimerization, which was significantly increased when treated with EGF (Figure 4A). However, when treated with pertuzumab (2C4), the fluorescence lifetime was seen to increase, in a manner similar to that in fixed cell experiment samples (Figure 4A). The EGFR/HER2 dimerization patterns correlated with the immunoprecipitation results (Figure 4B). As can be seen from the fluorescence images, we could only conclude whether EGFR receptors (upper panels, green) co-localized with HER2 receptors (lower panels, yellow) without the lifetime data (Figure 4C). However, our FRET assay could detect changes in EGFR/HER2 dimerization (Figure 4A). In combination with the fluorescence images, we could detect HER receptor dimerization at cellular level in paraffin-embedded cell pellets.

## DISCUSSION

Here, we present a proof-of-principle study demonstrating that we could use a pair of secondary antibodies from different species, conjugated to a pair of suitable FRET flurophores to assess EGFR/HER2 dimerization in human cell lines. This assay could be used to assess other HER dimerization pairings by changing the primary antibodies while using the same pair of flurophore-conjugated secondary antibodies. We demonstrated the sensitivity and specificity of the assay in detecting EGFR/HER2 dimerization in cell lines stimulated or treated with different doses of ligands and drugs. Most importantly, the assay is potentially applicable to tissue microarrays, as demonstrated by its effectiveness in paraffin-embedded cell pellet preparations. This will allow the dimerization states of tumor cells to be assessed *in-situ*. Thus, the assay can be assessed for its prognostic and predictive values in a clinical setting.

It has been claimed that assays other than FRET have the ability to assess protein interaction and specifically HER dimerization. These are based on the ligation of proteins, including Vera Tag [20] and Proximity Ligation Assay (PLA), which utilized DNA ligation [21]. However, none of these techniques can truly prove protein interaction although they can suggest it. Ligation assays can give positive results at a distance between substrates of 30-40 nm, which we would argue is very conclusive for confirming co-localization but not dimerization. Among all the techniques however, FRET is regarded as the gold standard technique to assess protein interaction since the distance between the determinants is <10 nm rather than 30-40 nm for both Vera Tag and proximity ligation assays [20-22]. Although there are many ways of determining FRET, methods based on the use of FLIM are the most sensitive in their ability to determine small changes in interaction distance and can thus be used as the basis for highly sensitive assays [22, 23]. The inverse sixth power distance dependence of FRET confers sensitivity to this assay, and the Förster Distance, at which half maximal FRET efficiency occurs, is typically ~5 nm [22, 23]. FRET induced changes in fluorescence lifetime are thus likely to represent direct interaction between receptors and have the potential advantage of accurately reflecting the dimerization of the receptors. It could be argued that by using the secondary antibody technique, we have increased the distance between the fluorophores and interfere with their interaction. This will decrease FRET efficiency and thus reduce the assay sensitivity. Therefore, it is possible that an even greater FRET efficiency would have been observed if primary conjugation was used. However, we have demonstrated our assay to be sensitive and specific in assessing EGFR/HER2 dimerization assay in various cell lines. Furthermore, the secondary antibody technique has the additional advantage of lower cost and thus potentially wider application to assess various protein interactions, compared with the primary conjugation approach.

Using the FRET technique, we are able to image the FRET process in single cells, establishing heterogeneity within the sample and are therefore able to identify trends, which might otherwise be overlooked in techniques that combine protein from many cells for analysis. Further to this, heterogeneity in dimerization within a single cell can be identified and thus the potential exists to explore the role of cellular compartmentalization HER signaling, which has been demonstrated to have great significance in acquired resistance to targeted therapies [24]. We believe that this assay has the potential to act as a prognostic test in HER2 and EGFR positive breast cancer although much more validation work is required. For example, it is well known that fluorescence lifetimes are affected by a multitude of factors (e.g. temperature, pH, oxygenation status etc.) [22, 23] and these may become limiting when small FRET-induced lifetime changes are to be determined. In the experiments described here this does not seem to be the case as experimental and preparation conditions were tightly controlled. Furthermore, application to paraffin-embedded patient tissue material remains to be validated; it may be challenging due to the presence of other fluorophores (e.g. endogenous auto-fluorescence), but the use of FRET fluorophores in the far-red portion of the spectrum may alleviate this. Suitably sensitive single photon counting detectors operating in the far-red are becoming available and could thus be applied. These assays may allow us to answer some of the unresolved questions regarding the unpredictable outcome of the currently available targeted treatments, such as Herceptin, and the role of HER dimerization in drug resistance.

## MATERIALS AND METHODS

### Materials and Cell Lines

A431 and SKBR3 cells were purchased form Cancer Research UK (CR-UK) and cultured in Dulbecco's Modified Eagle Medium (DMEM) with 10% fetal bovine serum and antibiotics. BT474 cells were cultured in Roswell Park Memorial Institute Medium (RPMI) with 10% fetal bovine serum and antibiotics. For these cells, 10μg/ml insulin was added to the medium when the cells were passaged. F4-IgG1, a mouse monoclonal antibody recognizing the cytoplasmic domain of EGFR, was obtained from CRUK London Research Institute. Anti-HER2 antibodies recognizing the intracellular residues were obtained from Cell Signaling Technology. Mouse and rabbit secondary antibodies conjugated to Alexa488 or Alexa546 were purchased from Invitrogen. HER ligands, EGF and heregulin were purchased from Sigma Aldrich. Gefitinib (Iressa) was purchased from Tocris and pertuzumab was kindly provided by Roche.

### Förster Resonance Energy Transfer experiments

Approximately, 30,000 cells were seeded onto cover slips in a 24-well plate and left to settle overnight. Cells were treated with drugs or ligands for specific times at 37 °C in culture media. After treatment, the staining protocol was performed at room temperature. Cells were washed with PBS and fixed with 4% paraformaldehyde (Pierce) in PBS for 10 minutes, then permeabilized with 0.2% Triton X-100 (Sigma-Aldrich) in PBS for 5 minutes before treatment for 10 minutes with 1 mg/ml sodium borohydride (Sigma-Aldrich) in PBS to quench the background fluorescence. Between steps, cells were washed twice with PBS. After quenching the background fluorescence, a blocking step was performed for 1 hour using 1% BSA (Sigma-Aldrich) in PBS. After blocking, primary antibodies (1:200 in blocking buffer) were added and allowed to bind for 2.5 hours. Cells were washed twice with PBS and Alexa-488 or Alexa-546 conjugated secondary antibodies (mouse or rabbit) (1:400 in blocking buffer) added for 90 minutes. Cells were washed twice with PBS and twice with water before mounting the cover slips on a microscope slide using Fluoromount-G (Southern Biotech).

For the cell pellet studies, 10^7^ A431 cells were embedded in paraffin. Embedded pellets were sliced by microtome and de-waxed. Antigen retrieval was performed, after which the protocol was used as described above.

### Fluorescence Lifetime Microscopy (FLIM)

FLIM and wide field fluorescence imaging were performed using a custom-built inverted microscope system operating in the single-photon excitation regime. FLIM was performed with excitation from a pulsed (~4 ps pulse width) super continuum laser source (SC450-M, Fianium Ltd., UK) operating at a repetition rate of 80 MHz and an optical filter set consisting of an excitation filter (Semrock FF01-470/22), dichromatic reflector (Semrock FF495Di02) and an emission filter (Semrock FF01-520/35) and a custom built laser scanning system. Emitted fluorescence light from the sample was collected through the emission filter by a photomultiplier tube (PMH-100-0, Becker & Hickl GmbH, Germany) in the de-scanned laser path and fluorescence lifetime information collected by a time-correlated single photon counting PCI board (SPC830, Becker & Hickl GmbH, Germany). Wide field fluorescence images were obtained with illumination from a metal halide arc lamp (Lumen200, Prior Ltd., UK) and detection with a 1344 × 1024 pixel CCD camera (ORCA-ER, Hamamatsu Photonics) at 160 nm/pixel resolution. To image Alexa 488 fluorescence a ‘FITC’ filter set was used (Excitation: 465-495 nm, Emission: 515-555 nm) and to image Alexa 546 a ‘TRITC’ filter set was used (Excitation: 528-552 nm, Emission 578-632 nm). The sample was illuminated and imaged through a 40x, 1.3 NA objective lens (Nikon S Fluor oil) in an epi-illumination configuration. For FRET analysis, pixel-by-pixel lifetime determination was utilised. The FLIM images were obtained and the outputs files consist of all the fitting parameters recorded for each image. These data can be analyzed to produce distinct lifetimes or a distribution of lifetimes across the image. The secondary conjugation preparation gives two separate fluorescence lifetimes. Thus, a bi-exponential fluorescence model was used to fit the data using in-house exponential fitting software (TRI2) utilising a Levenberg-Marquardt algorithm [25], modified to derive the maximum likelihood estimate [25], with the larger value interpreted as that of the Alexa 488 dye (Supplementary Figure 1). Pseudo-colour fluorescence lifetime maps were also produced using TRI2 [26].

### Immunoprecipitation

A431, BT474 and SKBR3 cells were grown to near confluence before lysis. Lysate was centrifuged for 10 minutes at maximum speed at 4°C and protein quantification was performed on the supernatant. Streptavidin-coated magnetic beads (Bio-Nobile) were incubated with biotin-conjugated HER2 or F4 antibodies (Conjugation Kit, Innova Biosciences) for two hours at room temperature [13]. After that, the bead-antibody complex was incubated with supernatant containing equal amounts of protein for 2 hours at room temperature. Bead complexes were collected and washed three times with PBS-Tween (0.2%). Next, 50 μl of 4× SDS with 10% β-mercaptoethanol was added to the bead complexes. Complexes were boiled for 10 min at 95 °C to elute protein from the beads. Samples were run on a SDS gel at 130 V. Proteins were transferred to a membrane using a semi-dry transfer at 12V for 2 hours. Membrane was blocked with 3% BSA in PBS-Tween (0.2%) for 1 hour. Primary antibodies were administered for 3 hours at room temperature in the blocking solution, followed by 4 washes with 1% milk in PBS-Tween. Secondary antibodies were added in 5% milk in PBS-Tween for an hour at room temperature. Four washes in 1% milk in PBS-Tween were performed before visualizing antibodies by an enhanced chemiluminescent (ECL) detection system (GE Healthcare).

### Statistical Analysis

Statistical analysis was performed using SPSS Version 18.0, including generation of box plots. ANOVA and the student's T-test were used to establish statistical significance between the means of different conditions.

## Supplementary Figure


