# Supplementary material for: Assessment of EGFR/HER2 dimerization by FRET-FLIM utilizing Alexa-conjugated secondary antibodies in relation to targeted therapies in cancers
Source: Oncotarget. 2011 Sep 8;2(9):728–36. doi: 10.18632/oncotarget.313 (PMC3248220; doi:10.18632/oncotarget.313)
Supplement: Supplementary file 1 [file oncotarget-02-728-s001.pdf]

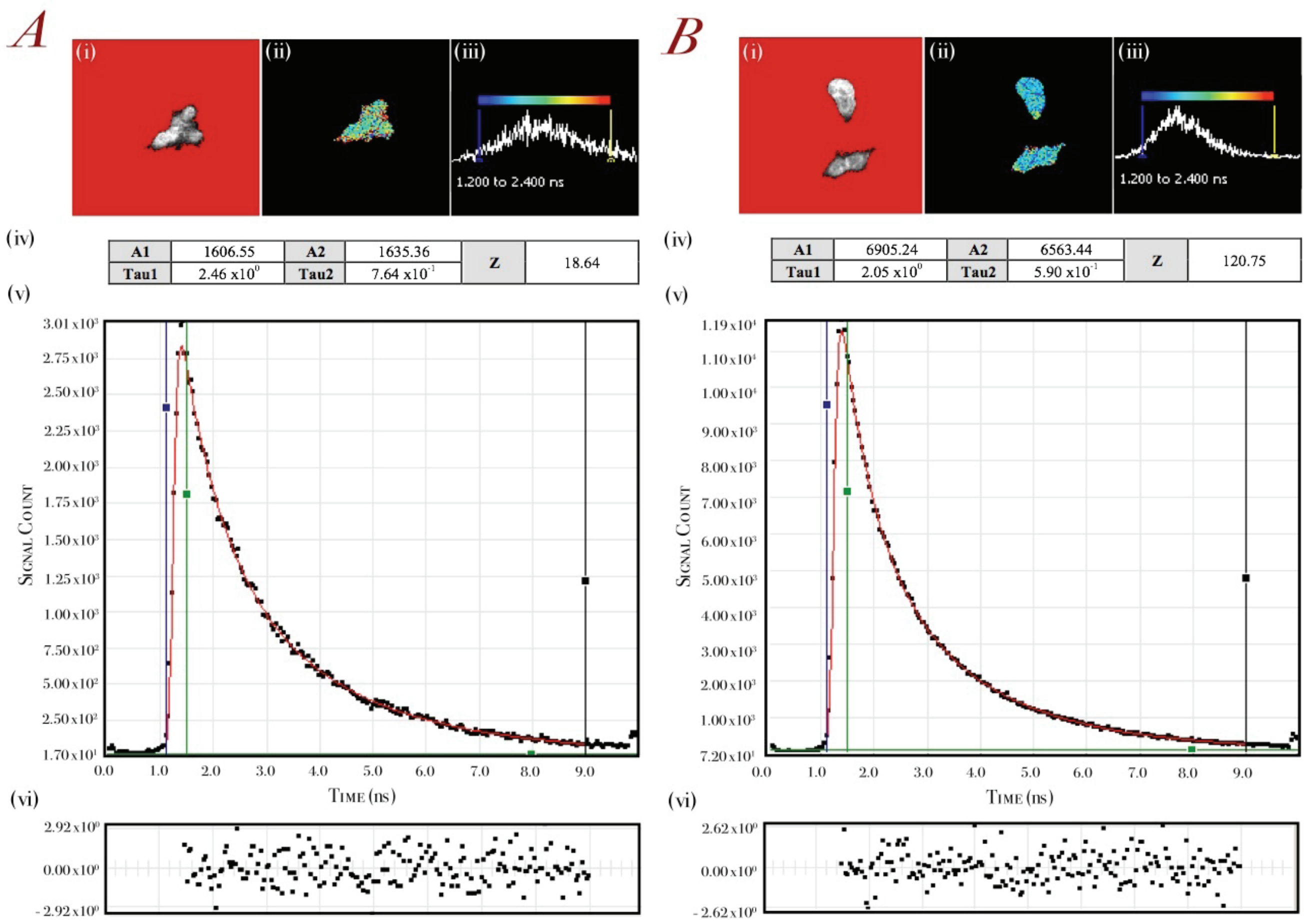

Supplementary Figure 1: Sample Time Resolved Analysis
Using TRI2 a bi-exponential fit was achieved utilizing a Levenburg-Marquardt algorithm, with a fit function f(t) = Z + A1 exp(-t/Tau1) + A2 exp(-t/Tau2), which was optimised for a maximum likelihood estimate (MLE). The fitted parameters were all free with the exception of X, which was restrained to be greater than zero. Here Tau1 and Tau2 represent the fitted slow and fast fluorescence lifetime components respectively in nanoseconds, while A1 and A2 represent their intensities in arbitrary units. **A.** An example of this analysis for a group of cells labelled with Donor antibodies alone and **B.** The same data for cells labelled with Donor and Acceptor antibodies pre-treated with EGF. In each case, (i) greyscale intensity images for each group of cells in which a brighter shade represents a greater number of photons; (ii) pseudo-colour images representing the pixel-by-pixel fluorescence lifetimes across the groups of cells; (iii) the corresponding distribution of lifetimes for the cells showing the pseudo-colour spectrum (1.2 ns to 2.4 ns) which relates to (ii); (iv) gives the fitted parameters in each case and (v) are examples of the data with the superimposed fitted bi-exponential curve applied to the data summed from all pixels within the field, and so a global analysis; (vi) shows the difference between the data and the fit, which has been weighted by the reciprocal of the square root of the fit.
